# The extracts of osteoblast developed from adipose-derived stem cell and its role in osteogenesis

**DOI:** 10.1186/s13018-024-04747-3

**Published:** 2024-04-22

**Authors:** Rattanawan Tangporncharoen, Atiruj Silathapanasakul, Patcharapa Tragoonlugkana, Chatchai Pruksapong, Tulyapruek Tawonsawatruk, Aungkura Supokawej

**Affiliations:** 1 Department of Clinical Microscopy, Faculty of Medical Technology, Mahidol University, Nakhon Pathom, 73170 Thailand; 2 Division of Plastic and Reconstructive Surgery, Department of Surgery, Pramongkutklao College of Medicine, Bangkok, 10400 Thailand; 3 Department of Orthopaedics, Faculty of Medicine, Ramathibodi Hospital, Mahidol University, Bangkok, 10400 Thailand

**Keywords:** Mesenchymal stem cell, Biologics, Cell therapy, Cell extracts, Cell-derived product, Bone regeneration, Osteogenesis, Osteoanabolic

## Abstract

Cell-based therapy has become an achievable choice in regenerative medicines, particularly for musculoskeletal disorders. Adipose-derived stem cells (ASCs) are an outstanding resource because of their ability and functions. Nevertheless, the use of cells for treatment comes with difficulties in operation and safety. The immunological barrier is also a major limitation of cell therapy, which can lead to unexpected results. Cell-derived products, such as cell extracts, have gained a lot of attention to overcome these limitations. The goal of this study was to optimize the production of ASC-osteoblast extracts as well as their involvement in osteogenesis. The extracts were prepared using a freeze–thaw method with varying temperatures and durations. Overall, osteogenic-associated proteins and osteoinductive potential of the extracts prepared from the osteogenic-induced ASCs were assessed. Our results demonstrated that the freeze–thaw approach is practicable for cell extracts production, with minor differences in temperature and duration having no effect on protein concentration. The ASC-osteoblast extracts contain a significant level of essential specialized proteins that promote osteogenicity. Hence, the freeze–thaw method is applicable for extract preparation and ASC-osteoblast extracts may be beneficial as an optional facilitating biologics in bone anabolic treatment and bone regeneration.

## Introduction

Cellular therapy is an upcoming strategy for various clinical applications, including degenerative diseases. Musculoskeletal degeneration, such as osteoarthritis or osteoporosis, affords a reasonable model for study owing to adverse medicinal effects and complications that remain problematic; even numerous licensed drugs are available [1–3]. It involves the transplantation of functional cells to replace and restore damaged tissues and organs. Mesenchymal stem/stromal cells (MSCs) are multipotent stem cells isolated from various sources (i.e., bone marrow, adipose tissue, umbilical cord blood, and placenta) which able to replicate infinitely and commit into specific cell lineages, such as osteocytes, adipocytes, and chondrocytes [4, 5]. In addition, MSCs also secrete a broad of cytokines, growth factors, and chemokines, which possess immunomodulatory functions [6, 7]. There are many types of adult stem cells containing the MSCs’ characteristics such as bone marrow-derived MSCs, umbilical cord-derived MSCs, menstrual blood stem cells, dental pulp stem cells, and adipose-derived stem cells (ASCs) [8]. Beyond their potential, ASCs are accessible through a minimally invasive process, are easily isolated, and can be obtained in large numbers [9, 10]. ASCs have become an appealing cell for application in cell-based regenerative medicine and have been used in numerous preclinical and clinical applications [9–12]. An injection of autologous ASCs in symptomatic knee osteoarthritis patients resulted in clinically significant pain relief and functional improvement, with no serious adverse events [13, 14]. Applying ASCs to the rat femoral defect model accelerates new bone formation [15], as does a case report of a severe head injury with multifragment calvarial fractures [16].

In spite of their therapeutic efficacy, multiple processes of cell production are considered major factors influencing cellular properties. The living cells must be aware of cell origin, cell number, cell quality, immunogenicity, tumorigenicity, and donor compatibility [17–19]. Cell-derived products, including extracellular vesicles, cell lysate, and conditioned medium, which contain many bioactive substances, are becoming increasingly popular in research studies and clinical trials. Cell extracts are as medicinally effective as whole-cell, lending credence to the concept that cells might respond by releasing internally performed biomolecules [20]. Cell extracts consist of soluble intracellular components, such as growth factors, chemokines, and cytokines, that are lysed from cells and have a lower immunogenic and tumorigenic risk [21, 22]. An intravenous delivery of bone marrow cell extract in the presence or absence of a calcium phosphate scaffold can promote new bone formation in rats with osteoradionecrosis without triggering an immunological response [23]. Cell extracts from bone marrow, adipose tissue, and spleen can correct salivary gland (SG) function in irradiated-injured SG mice by increasing cell proliferation, protecting acinar cells, blood vessels, and parasympathetic nerves and restoring saliva secretion [24, 25]. As well, pretreatment of human adipose tissue with membrane-free stem cell extract reduced ischemic brain injury and blood–brain barrier disruption in mice with ischemic stroke. However, it downregulated proinflammatory cytokines expression and decreased cell death [26].

Even though their applications and benefits are well-known, the technique for producing cell extracts is not well established and lacks standardization. The preparation is varied upon materials and methods among laboratories [24, 26–31]. Additionally, enhancing the regeneration potential for specific purposes is challenging. Preconditioning, genetic modification and tissue engineering are the most common strategies [22]. MSCs preconditioned with proinflammatory cytokine tumor necrosis factor alpha (TNF-α) plus lipopolysaccharide [32] or TNF-α alone [33] have increased osteogenic differentiation including alkaline phosphate activity and matrix mineralization. The stimulation of ASCs with TNF-α potentiated their exosome efficiency to promote proliferation and osteogenic differentiation in osteoblastic cells [34]. Osteogenic-differentiated ASCs are superior to undifferentiated ASCs in aspects of angiogenicity and osteogenicity for bone allografts [35, 36]. Priming approaches can empower therapeutic efficacy, in particular when modulating cell fate [37] and performance [38–41]. Thereby, it is beneficial for direct preconditioning of cell phenotypes in therapeutically desirable ways.

This study aimed to optimize the freeze–thaw production of cell extracts and investigate the role of extracts prepared from osteogenic-induced ASCs (ASC-osteoblast extracts) on in vitro mesenchymal-osteoblastic differentiation. To determine the best preparation method, the extracts were prepared using the freeze–thaw technique under several conditions differing in temperature and duration. The expression of osteogenic genes and bone morphogenic protein families were determined on the indicated day during ASC-osteoblastic differentiation. The ASC-osteoblast extracts were prepared on the indicated day and then applied as a supplement in osteogenic differentiation. The findings of this study would strengthen the future use of the extracts prepared from osteogenic-induced ASCs as a cell-derived product in bone regeneration treatment.

## Materials and methods

### ASCs isolation and expansion

ASCs were isolated from fresh adipose tissue obtained from lipoaspiration from healthy donors with written informed consent. The process of cell isolation was briefly as follows: aspirated adipose tissue was washed with penicillin–streptomycin added phosphate buffer saline (PBS) to remove excess oil and blood. The samples were then diluted with type II collagenase solution at a 5:1 ratio and incubated at 37 °C for an hour. Samples were centrifuged, vigorously mixed and repeat centrifugation. The cell pellet was mixed with red blood cell (RBC) lysis buffer at room temperature and washed twice with DMEM-LG. Cells were seeded into a 6 well plate, cultured with growth medium containing DMEM-LG (Gibco, USA) supplemented with 10% fetal bovine serum (FBS; Sigma-Aldrich, USA), 1% GlutaMAX™ (Gibco, USA), and 1% Penicillin–Streptomycin (Gibco, USA) and maintained in 37 °C, 5% CO_2_ with humidified condition. After 72 h, non-adherent cells were removed. The medium was changed every 3–4 days and subpassaged when reach to 80–90% confluency.

### Osteogenic differentiation

To induce ASCs to differentiate into the osteogenic lineage, cells were seeded in 100 mm dish at a density of 1.2 × 10^6^ cells per dish and cultured overnight in 37 °C, 5% CO_2_ with humidified condition. After 24 h, growth medium was changed to osteogenic differentiation medium (ODM) containing growth medium supplemented with 0.1 µM dexamethasone (Sigma Aldrich, USA), 50 µg/ml ascorbic acid (Sigma Aldrich, USA), and 10 mM β-glycerophosphate (Merck KGaA, Germany). The medium was changed every 3 days. During differentiation, osteogenic-induced ASCs on the designated day were taken for gene expression study (N = 4) and cell extracts preparation.

### Cell extracts preparation

Freeze-thawing technique were employed for preparing the extracts. Cells were trypsinized and washed twice with PBS prior to centrifuge. The pellet was resuspended in normal saline solution (NSS) and adjusted in a concentration of 4000 cells/µl. Cell suspension was frozen for an hour and immediately thawed at 37 °C. The process was repeated 3 times. After the 3rd cycle, protease inhibitor cocktail (Calbiochem, USA) was added to the whole cell lysate, which was subsequently centrifuged at 12,000 g at 4 °C. Supernatant was collected and stored at -20 °C.

The ASC-osteoblast extracts were performed using osteogenic-differentiated ASCs on certain day 3 (EX-D3), 7 (EX-D7), and 10 (EX-D10) for protein analysis (N = 4), as did undifferentiated ASCs employed as a control (EX-CT). In addition, the effect of extracts on differentiation was explored. Three numbers of ASC-osteoblast extracts were mixed up and added to ODM at the concentration of 10 ug/ml for treating ASCs (N = 3).

### RNA extraction and qRT-PCR

Cells were harvested in TRIzol™ reagent (Thermo Fisher Scientific, USA) and total RNA was extracted using Direct-zol™ RNA MiniPrep Kits (Zymo Research, USA). The concentration and purity of RNA were detected by a NanoDrop™ 2000 spectrophotometer (Thermo Fisher Scientific, USA). cDNA was synthesized according to the instruction of iScript RT Supermix (Bio-Rad, USA). Quantitative reverse transcription polymerase chain reaction (qRT-PCR) was performed on CFX96 Touch Real-Time PCR System (Bio-Rad, USA) using KAPA SYBR® FAST qPCR Master Mix (Kapa Biosystems, USA). The osteogenic-related genes examined were runt-related transcription factor 2 (*RUNX2*), osterix (*OSX*), and osteopontin (*OPN*). The housekeeping gene glyceraldehyde-3-phosphate dehydrogenase (*GAPDH*) was utilized to standardize RNA expression levels. The gene specific primers were listed.

**Table:** 

Gene	Primer sequence
*RUNX2*	Forward: 5′-AACCCAGAAGGCACAGACAG-3′
	Reverse: 5′-GCCTGGGGTCTGTAATCTGA-3′
*OSX*	Forward: 5′-TGCTTGAGGAGGAAGTTCAC-3′
	Reverse: 5′-CTGCTTTGCCCAGAGTTGTT-3′
*OPN*	Forward: 5′-ACAGCCAGGACTCCATTGAC-3′
	Reverse: 5′-GGGGACAACTGGAGTGAAAA-3′
*GAPDH*	Forward: 5′- CAACTACATGGTTTACATGTTCCAA-3′
	Reverse: 5′- CAGCCTTCTCCATGGTGGT-3′

### Bradford assay

The amount of total protein in cell extracts was determined by Bradford assay. Bradford reagent containing 0.01% (w/v) Coomassie blue G-250 (Merck KGaA, Germany), 4.75% (w/v) ethanol (Merck KGaA, Germany), and 8.5% (w/v) phosphoric acid (Merck KGaA, Germany) was mixed with the extracts and incubated at room temperature in the dark for 10 min. The absorbance was measured at 595 nm. Protein concentration in the samples was calculated by comparison to bovine serum albumin (BSA) standard curve.

### Propidium iodide (PI) staining

For assessment of cell components in cell extracts, the extracts were stained with PI adapted from the manufacturer’s instructions. 25 µl of samples was mixed with 5 µl of PI (Component no. 51-66211E; BD Biosciences, USA) and incubated at room temperature in the dark for 15 min. The samples were examined with FACS Canto II flow cytometer (BD Biosciences, USA) and Floreada.io software (https://floreada.io/).

### Enzyme-linked immunosorbent assay (ELISA)

Growth factors presented in ASC-osteoblast extracts were quantified by a sandwich enzyme immunoassay ELISA kit (ELK biotechnology, China). The levels of BMP4 (ELK7500), BMP6 (ELK1860), BMP7 (ELK2635), TGFb1 (ELK1185) and TGFb3 (ELK3721) were analyzed in accordance with the manufacturer’s instructions. The absorbance was measured with a Multiskan SkyHigh Microplate Spectrophotometer (Thermo Fisher Scientific, USA) and compared to a standard curve to determine the concentration.

For the determination of BMP2, an indirect ELISA assay was established. Samples and working standard (Recombinant Human/Mouse/Rat BMP-2; BioLegend, USA) diluted in 0.1 M NaHCO_3_ were applied to 96-well half area microplate (Corning, USA) and incubated at 4 °C overnight. The solution in each well was removed, blocking buffer containing PBS supplemented with 1% BSA (Merck KGaA, Germany) was added, and the plate was incubated at room temperature for 2 h. After incubation, the solution was discarded and incubated with anti-BMP2 antibody (Lot no. G9231-4B12; Sigma Aldrich, USA) for an hour. HRP-linked antibody (Cell Signaling Technology, USA) was mixed up and incubated again. In each phase, the solution was aspirated and washed 3 times with 0.1% Tween in PBS. Finally, TMB solution (Merck KGaA, Germany) was added and incubated in the dark until the color changed into blue, followed by the addition of 1 N HCl to stop the reaction. Absorbance was measured at 450 nm.

### Statistical analysis

Data are presented as mean and standard error of the mean (SEM). Statistical analysis was analyzed using Mann–Whitney U Test and *p*-value < 0.05 were considered as statistically significant.

## Result

### The determination of osteogenic characteristics during mesenchymal-osteoblastic differentiation

**Fig. 1:**
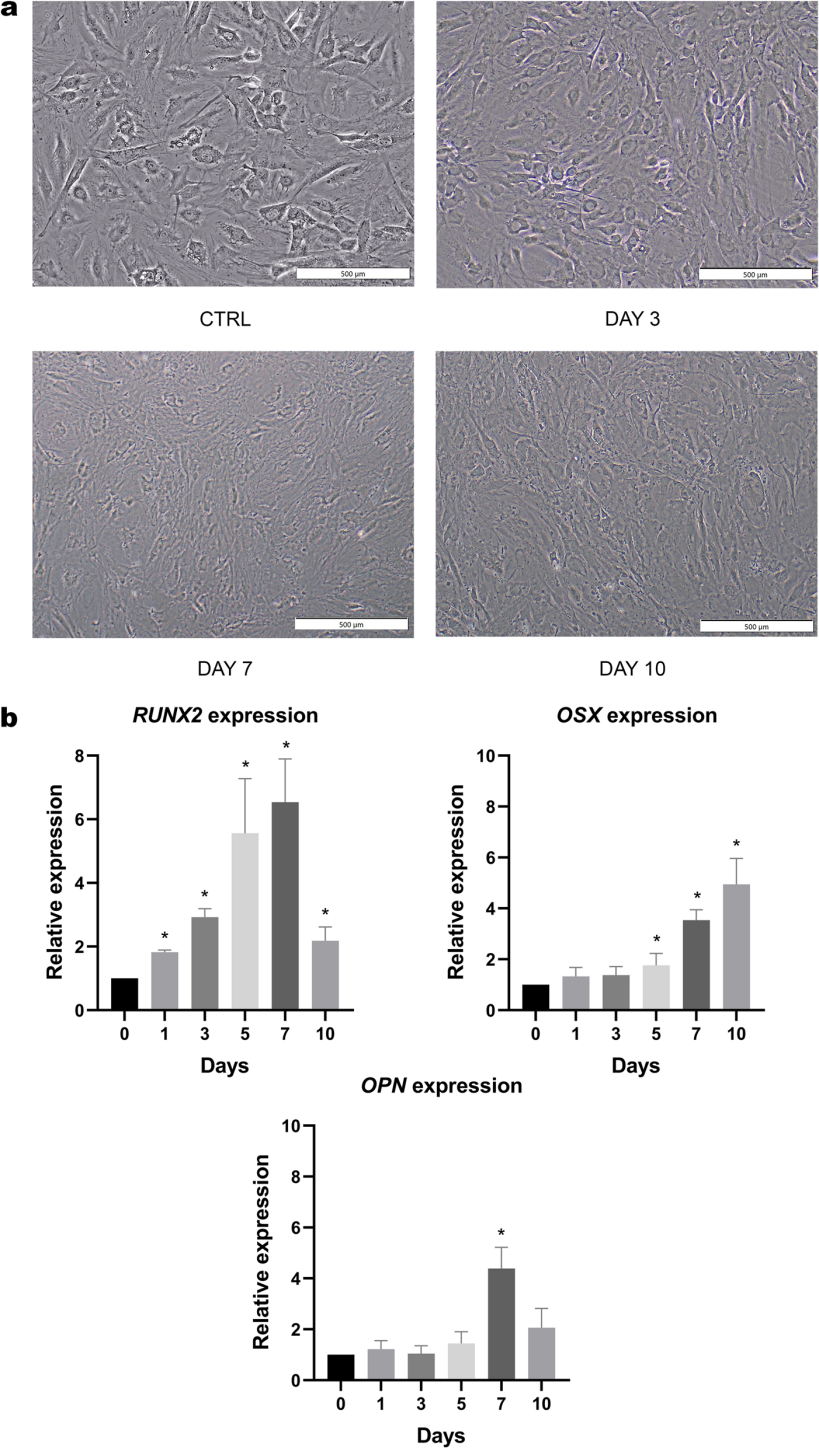
The morphology of ASCs was observed during osteogenic differentiation on days 3, 7, and 10 (**a**). The expression of osteogenic markers (**b**), including *RUNX2*, *OSX* and *OPN* were presented as a relative expression compared with that of control during 10 days of osteogenic differentiation. All data were shown as mean ± SEM. *p*-value < 0.05 is identified as a statistically significant difference. (N = 4)

ASCs were cultured for 10 days with osteogenic differentiation medium and harvested on day 1, 3, 5, 7, and 10. Cell morphology of ASCs during mesenchymal-osteoblastic differentiation had been observed by inverted microscope. The morphology appeared as spindle shaped with increasing cell density (Fig. 1a). The expression patterns of osteogenic related genes (*RUNX2*, *OSX,* and *OPN*) had been studied by qRT-PCR. The transcriptional level of *RUNX2* increased significantly during differentiation, gradually rising from day 1 to day 7, though it noticeably decreased on day 10. In the meanwhile, the expression of *OPN* was significantly upregulated on day 7 and initially reduced on day 10. Contrastingly, mRNA expression of *OSX*, an osteoblast-specific transcriptional factor, started to elevate on day 5 and continued to increase (Fig. 1b). An alteration in gene expression patterns under differentiation condition may govern cell phases and protein expression.

### The preparation of cell extracts

**Fig. 2:**
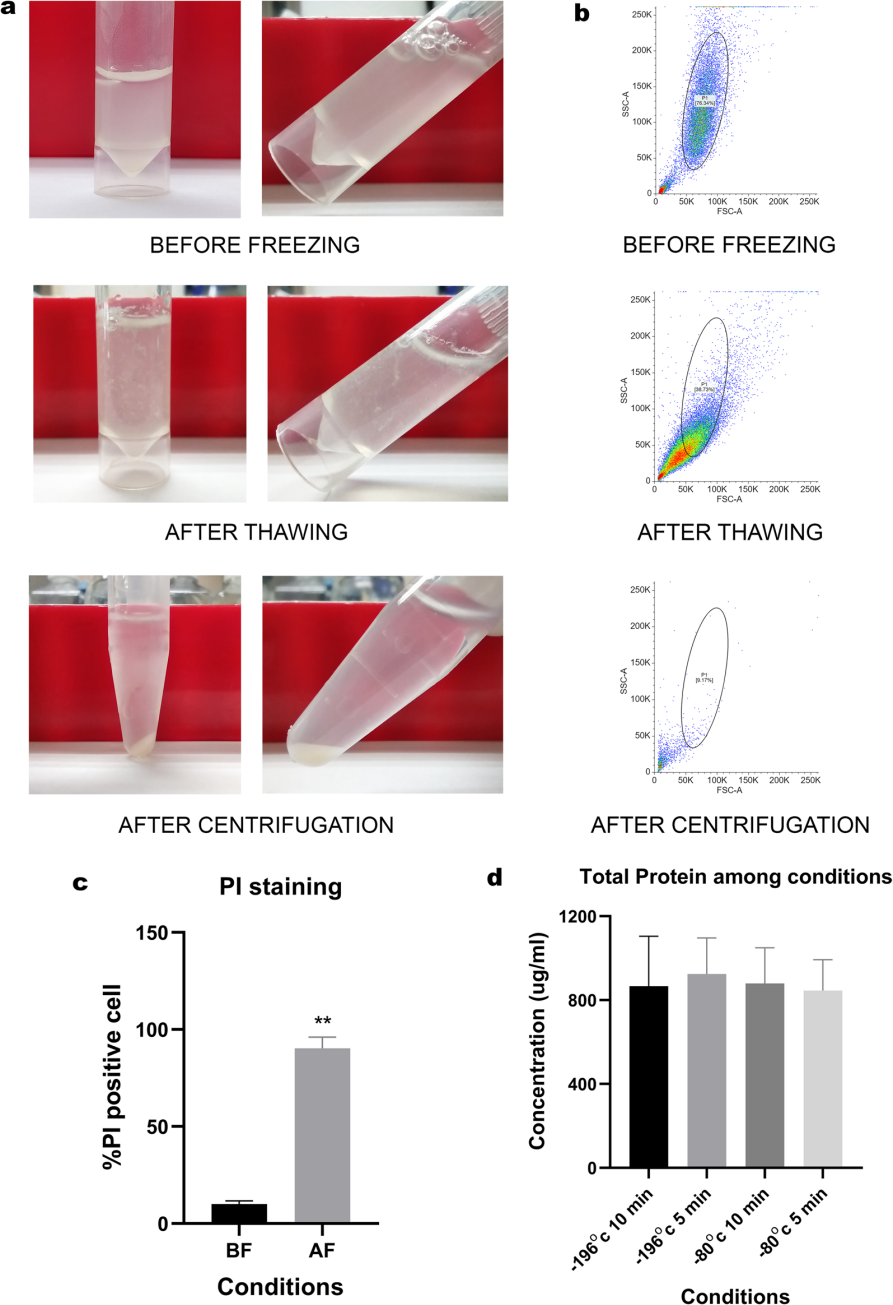
Cell extracts were produced among 4 conditions. The appearance of whole cell lysate was presented for all phases of preparation (**a**). Flow cytometry analysis and propidium iodide (PI) staining of the extracts before and after 3 freeze–thaw cycles as well as after centrifugation. The patterns were represented as dot plot (**b**). Quantitative analysis of PI positive staining before (BF) and after (AF) freeze–thaw cycle was shown in bar chart (**c**). The concentration of protein in cell extracts were demonstrated comparing between conditions (**d**). All data were shown as mean ± SEM. (N = 3)

Thermal lysis is a simple and potential method for cell lysis technique. Freeze–thaw cycle causes the formation and melting of ice crystals, which breakdown cell membranes. Herein, temperature and duration are two critical factors that help in lysate quality. The preparation of cell extracts was conducted at four different conditions, varied in chilling temperatures (− 80 °C and − 196 °C) and defrosting periods (5 and 10 min). Therefore, the extracts were subjected to the following regulation: (1) − 196 °C freezing with 10 min of thawing, (2) − 196 °C freezing with 5 min of thawing, (3) − 80 °C freezing with 10 min of thawing, and (4) − 80 °C freezing with 5 min of thawing. The repetitive cycle of cell lysate caused cells to have more rupture, and almost all had been fragmented at the last cycle. Cell debris and nuclear components were also removed after centrifugation (Fig. 2a). Similarly, flow cytometry analysis indicated that after freeze–thaw, the cell population decreased in size and numbers. Total cell counts were scaled down from 75.39 ± 1.79 to 37.63 ± 3.88%, whereas the percentage of PI-positive staining raised to 90.29 ± 4.69% and subsequently gone by centrifugation (Fig. 2b and c). The amount of total protein showed similar among all conditions (Fig. 2d and Table 1). Therefore, a setting at − 80 degrees Celsius freezing with a 5-min warm-up was employed in this study due to the short duration of cell extracts preparation.

**Table 1:** Concentration of total protein in cell extracts

Conditions	Concentration (µg/ml)			
	− 196 °C 10 min	− 196 °C 5 min	− 80 °C 10 min	− 80 °C 5 min
N = 1	1291.14	1205.43	1155.43	1119.71
N = 2	468.29	612.57	569.71	619.71
N = 3	841.14	955.43	912.57	798.29
Mean ± SEM	866.86 ± 194.23	924.48 ± 140.31	879.24 ± 138.72	845.90 ± 119.44

### The expression of osteogenic associated growth factors during mesenchymal-osteoblastic differentiation

**Fig. 3:**
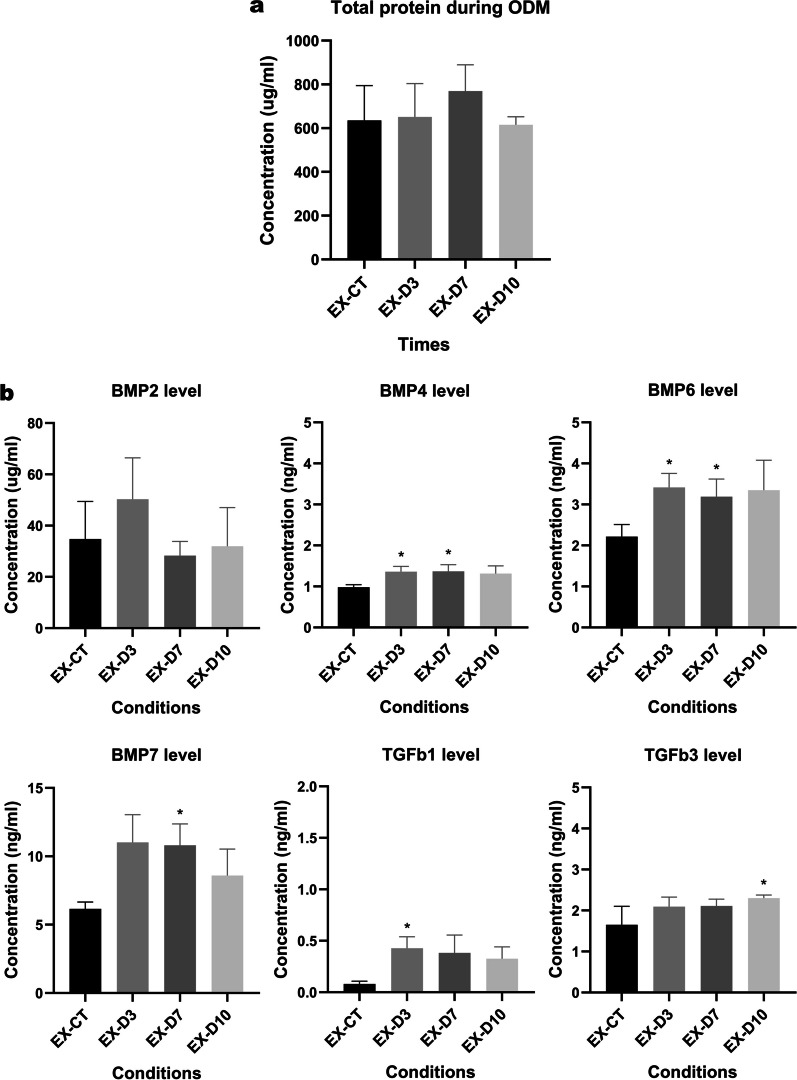
Protein concentration of ASC-osteoblast extracts after ASCs were cultured in ODM for 10 days (**a**) and osteogenic-associated growth factors, including BMP2, BMP4, BMP6, BMP7, TGFb1 and TGFb3 (**b**). Data were presented in the bar charts, and undifferentiated cell extracts were used as a control. All data were shown as mean ± SEM. *p*-value < 0.05 is identified as a statistically significant difference. (N = 4)

ASCs were grown under osteogenic induction condition for 10 days prior to cell extracts production. ASC-osteoblast extracts were made up of osteogenic-induced ASCs at specified day 3 (EX-D3), 7 (EX-D7), and 10 (EX-D10), with the extracts of ASCs cultured in growth medium (EX-CT) being as a control. Additionally, total protein and relevant growth factors in the extracts were determined by Bradford assay and ELISA. The results revealed a slightly increased protein level on day 7 (Fig. 3a). Concurrently, the levels of six osteogenic-related growth factors including BMP2, BMP4, BMP6, BMP7, TGFb1 and TGFb3 were significantly greater than those in the undifferentiated extracts control group, except for BMP2, that was not statistically significant difference (Fig. 3b). For ASC-osteoblast extracts on day 3 (EX-D3), almost all exhibited the highest concentration, which was stably maintained until day 10, except for BMP2 and BMP7 that decreased at day 7 and 10 respectively. Taken together, these findings indicate that ASC-osteoblast extracts contain important osteogenic-associated growth factors and that the extracts of osteogenic-induced cells may improve the expression level of those factors.

### An involvement of ASC-osteoblast extracts during mesenchymal-osteoblastic differentiation

**Fig. 4:**
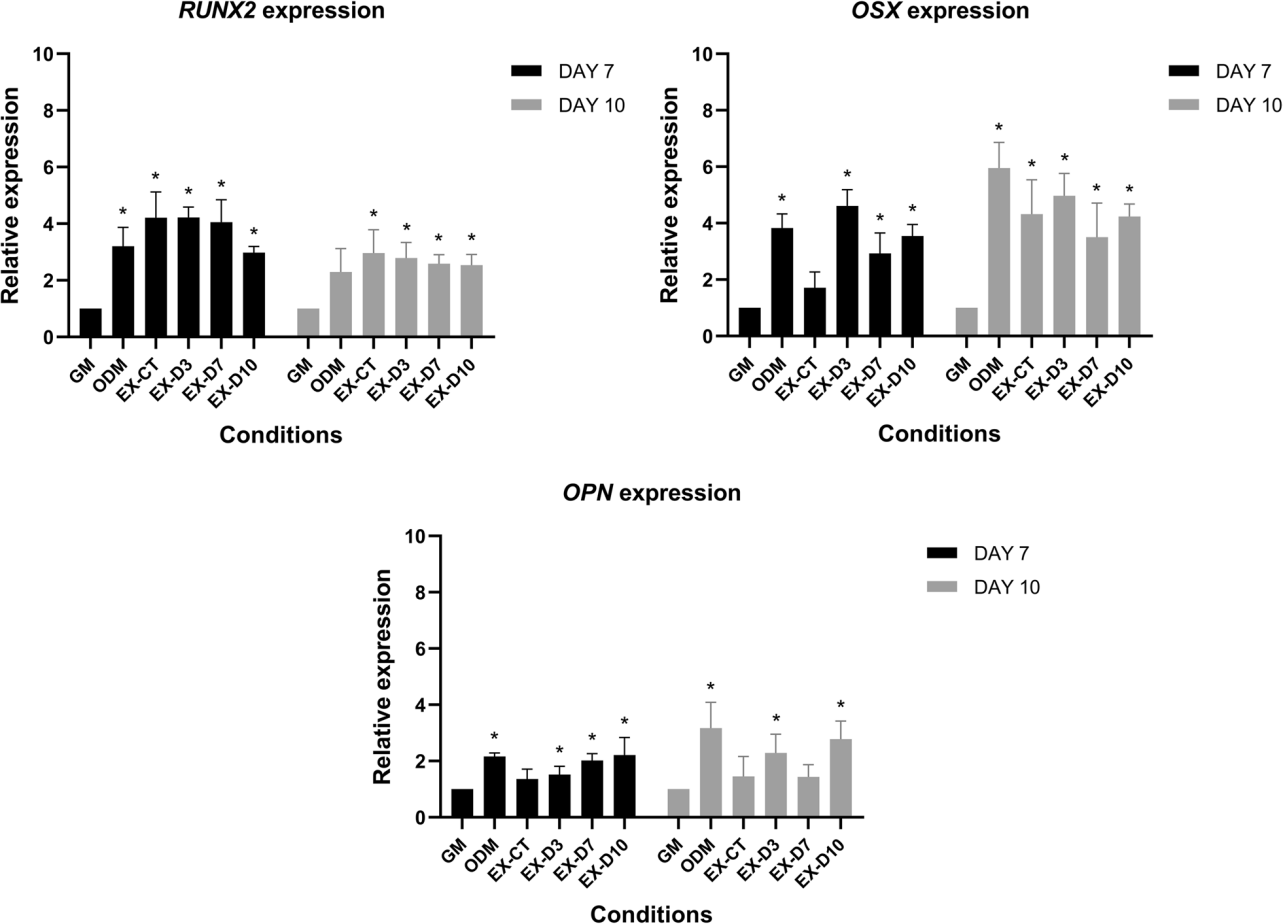
The expression of osteogenic-related genes, including *RUNX2*, *OSX* and *OPN* at day 7 and 10 after 10 days of 10 ug/ml ASC-osteoblast extracts treated under osteogenic induction were presented as a relative expression compared with that of control group. All data were shown as mean ± SEM. *p*-value < 0.05 is identified as a statistically significant difference. (N = 3)

To demonstrate whether ASC-osteoblast extracts could assist in cell differentiation. The extracts were produced from normal ASCs (EX-CT) and osteogenic-induced ASCs on day 3 (EX-D3), 7 (EX-D7) and 10 (EX-D10). The three of extracts within the same condition were pooled together before treating ASCs under osteogenic differentiation condition for 10 days, with ASCs grown in growth medium (GM) serving as a control. Cells were harvested and RNA was collected on day 7 and 10. qRT-PCR was employed to investigated 3 osteogenic-associated genes: *RUNX2*, *OSX*, and *OPN*. All transcriptional levels were found to be significantly higher than those in the growth medium control (Fig. 4). The mRNA expression of *RUNX2* in the presence of ASC-osteoblast extracts was greater than that in the absence of treatment (ODM), but it appeared to be comparable across ASC-osteoblast extracts conditions. In contrast, the expression of *OSX* and *OPN* seemed to be higher after co-culture with ASC-osteoblast extracts of osteogenic-induced ASCs than co-culture with normal ASC extracts.

## Discussion

Biologics are a wide collection of products that include vaccines, growth factors, antibodies, cell and gene therapy. Cell extracts are under interesting aimed for using as a biological-derived cell free product. To prepare cell extracts, several methods, such as mechanical, physical, chemical and biological techniques, have been established for lysing cells. Each technique applies a unique approach to breaking down the cell membrane and has its own set of advantages and disadvantages [42, 43]. The freeze–thaw method is a type of physical technique, which creates thermodynamic instability and causes the disruption of cell membrane. This method is preferred because it is inexpensive, easy to implement, and requires no reagents or equipment. Previous studies demonstrated plenty of freeze–thaw protocols. For example, Fang et al. applied − 80 °C chilling with 37 °C melting to obtain cell extracts [25]. Song et al. incubated MSC on dry ice rather than in a − 80 °C freezer [29, 44]. Hu et al. conducted embryonic germ cell extracts by repeatedly storing and removing them in liquid nitrogen [45]. Peng et al. performed freeze–thaw cycles using liquid nitrogen and a 37 °C water bath [46]. This study investigated temperature and duration parameters during the thermal lysis procedure. Cell extracts were set up at various freezing temperatures (− 80 °C or liquid nitrogen) and thawing periods (5 or 10 min) but with the same cycling numbers. The total protein concentration in all extracts was comparable, ranging between 800 and 900 µg/ml. The concentration of our extracts correlated with that of ADSC soup in the work of Fang et al., which was roughly fivefold diluted because the density of cell suspension was lower [25]. According to the results, frosting between − 80 °C and liquid nitrogen (− 196 °C) seemed to have negligible effects on protein quantity, as did an extended defrosting time. This is contrary to previous findings in which temperatures of − 80 °C and liquid nitrogen were found to differ in terms of protein yield, with freezing in liquid nitrogen obtaining larger yields [47]. The research of Tan et al. also showed that a decrease in freezing temperature and prolonged thawing time can result in more protein [48]. However, the structural differences between prokaryotic and eukaryotic cell membranes may affect the lysis method. Additionally, we reported that three repeated freeze–thaw cycles can help in cell breakdown, and previous studies supported that 2–3 repeated cycles were appropriate [49].

Apart from the preparation process, cell modification may exert protein concentration. During osteogenesis, ASCs develop into osteoblasts, which altered gene and protein expression. This study determined the expression of osteogenic-related genes during mesenchymal-osteoblastic differentiation. The transcriptional levels of *RUNX2*, *OSX*, and *OPN* changed depending on the cell stage. The levels progressively increased in the initial phase before dropping in the end, except for *OSX*, which kept going. The expression pattern resembled that of a previous study on the fate of MSCs undergoing osteogenic lineage differentiation [50, 51]. Likewise, osteoinductive proteins have been explored. Compared to undifferentiated ASC-osteoblast extracts, differentiated ASC-osteoblast extracts had significantly higher levels of growth factors, including bone morphogenic proteins (BMP) and transforming growth factor beta subfamily (TGFb). The transformation of ASCs into osteoblasts may result in the transcription and translation of associated proteins. BMPs are biomolecules that belong to the transforming growth factor beta (TGFb) superfamily. BMPs involved in bone development, especially BMP2, BMP4, BMP6, and BMP7 [52–55]. Meanwhile, TGFb1 and TGFb3, which are members of the TGFb subfamily, also displayed with bone forming abilities [55–58].

Although BMPs and TGFb themselves exhibit satisfactory functions, combinations of these two or multiple BMPs have been researched. Kaito et al. reported that the combination of BMP2 and BMP7 heterodimers is a stronger inducer of bone regeneration than individual homodimers in a rat spinal fusion model [59, 60]. BMP4 and BMP6 might synergistically induce osteoblast differentiation of C2C12 cells, resulting in higher alkaline phosphatase activity than that of only one of these BMPs [61]. Co-transfection of BMP2/TGFb3 into rabbit bone marrow-derived mesenchymal stem cells (rBMSCs) enhanced osteogenic transformation, as indicated by elevated ALP activity, osteogenic-specific genes expression and calcium deposition [62]. Similarly, a combination of BMP2- and TGF-loaded scaffold promoted greater bone growth than either growth factor-free or solely BMP2-loaded groups [63, 64]. A mixture of BMP2, VEGF, and TGFb1 can considerably increase osteogenic differentiation, which is linked to a time-effect relationship [65]. As mentioned before, coupling growth factors might have a synergistic influence. The extracts are likely to be a cocktail of essential growth factors. Herein, the role of ASC-osteoblast extracts in mesenchymal-osteoblastic differentiation was investigated. ASC-osteoblast extracts drove cell differentiation by stimulating the expression of osteogenic-specific genes. Our findings were consistent with previous research showing that the extract of concentrated growth factor (CGF-e) enhanced differentiation and mineralization of MC3T3-E1 cells and accelerated bone formation in rat cranial defect model [66]. According to Saito et al. and Michel et al., cell extracts possess a therapeutic effect upon bone regeneration. Human umbilical cord extracts improved the functional abnormalities of bone marrow-derived MSC from ovariectomized rats [67]. An injection of bone marrow cell extract can restore irradiated bone without an immune response activation [23].

## Conclusion

In conclusion, the present study demonstrated the following: (i) The freeze–thaw method is practicable for cell extracts production in clinical laboratory using standard supplies. (ii) The induction of cells into a particular lineage may influence the expression of essential specialized proteins. (iii) ASC-osteoblast extracts consist of several types of growth factors that promote osteogenic-related gene expression. Actually, our study was limited to a single cell source and a reproducible number; other sources, such as bone marrow or umbilical cord should be reviewed. It ought to also be carried out for further research in points of immunological reaction, biomaterial supplementation, and storage effect.

## Data Availability

Floreada.io software is accessible (https://floreada.io/).

## Abbreviations

ASC: Adipose-derived stem cell
*RUNX2*: Runt-related transcription factor 2
*OSX*: Osterix
*OPN*: Osteopontin
BMP: Bone morphogenic protein
TGFb: Transforming growth factor-beta
